# Patient-Reported Outcomes From Phase III Neoadjuvant Systemic Trial Comparing Neoadjuvant Chemotherapy With Neoadjuvant Endocrine Therapy in Pre-Menopausal Patients With Estrogen Receptor-Positive and HER2-Negative, Lymph Node-Positive Breast Cancer

**DOI:** 10.3389/fonc.2021.608207

**Published:** 2021-07-02

**Authors:** Sungchan Gwark, Sei Hyun Ahn, Woo Chul Noh, Eun Sook Lee, Yongsik Jung, Lee Su Kim, Wonshik Han, Seok Jin Nam, Gyungyub Gong, Seon-Ok Kim, Hee Jeong Kim

**Affiliations:** ^1^ Department of Surgery, College of Medicine, Asan Medical Center, University of Ulsan, Seoul, South Korea; ^2^ Department of Surgery, Korea Cancer Center Hospital, Korea Institute of Radiological and Medical Sciences, Seoul, South Korea; ^3^ Department of Surgery, Center for Breast Cancer, Research and Institute and Hospital, National Cancer Center, Goyang, South Korea; ^4^ Department of Surgery, School of Medicine, Ajou University, Suwon, South Korea; ^5^ Division of Breast and Endocrine Surgery, Hallym University Sacred Heart Hospital, Hallym University, Anyang, South Korea; ^6^ Department of Surgery and Cancer Research Institute, College of Medicine, Seoul National University, Seoul, South Korea; ^7^ Department of Surgery, School of Medicine, Samsung Medical Center, Sungkyunkwan University, Seoul, South Korea; ^8^ Department of Pathology, College of Medicine, Asan Medical Center, University of Ulsan, Seoul, South Korea; ^9^ Department of Clinical Epidemiology and Biostatistics, Asan Medical Center, Seoul, South Korea

**Keywords:** quality of life, neoadjuvant chemotherapy, neoadjuvant endocrine therapy, patient-reported outcomes, Neoadjuvant stusdy of chemotherapy versus Endocrine therapy in premenopausal patient with hormone responsive, HER2-negative, lymph node-positive breaST cancer (NEST)

## Abstract

We aimed to evaluate the patient-reported outcomes (PROs) in a prospective phase III clinical trial, comparing neoadjuvant endocrine therapy (NET) with conventional neoadjuvant chemotherapy (NCT) in patients with hormone status positive, lymph node-positive premenopausal breast cancer (NCT01622361). The patients were randomized prospectively to either 24 weeks of NCT with adriamycin plus cyclophosphamide followed by taxane or NET with gonadotropin-releasing hormone agonist and tamoxifen. The patients were examined at the surgery unit of a large tertiary care hospital with a comprehensive cancer center. PROs were assessed on the first day of the trial (day 1, baseline) and at the end of treatment, using the breast cancer module of the European Organisation for Research and Treatment of Cancer Quality of Life Questionnaire Core 23 (EORTC QLQ BR23). One hundred and eighty-seven patients were randomly assigned to chemotherapy (n=95) or endocrine therapy (n=92), and 174 patients completed 24 weeks of the neoadjuvant treatment period (n=87, in each group). Baseline scores were similar between the groups. After treatment, there were no statistically significant differences in the function scales, including body image, sexual functioning, and sexual enjoyment between the groups, although the endocrine treatment group showed a significant improvement in the future perspective (hazard ratio, 8.3; 95% confidence interval, 1.72–18.38; P = 0.021). Similarly, there were no statistically significant differences in the symptom scales between the groups, including adverse effects of systemic therapy, breast symptoms, arm symptoms, and upset about hair loss. In conclusion, overall PROs were similar in both treatment groups, except for “future perspective,” which was significantly better in the NET group than in the NCT group.

**Clinical Trial Registration:**

ClinicalTrials.Gov, identifier NCT01622361.

## Introduction

Neoadjuvant chemotherapy (NCT) is becoming a more common treatment of choice for locally advanced breast cancer patients. Down-staging could lead to a lower extent of surgery, e.g., an increase in the breast conservation rate and a better cosmetic outcome (1–3). However, the adverse effects caused by chemotherapy for breast cancer have both short- and long-term consequences, and the frequency, duration, and severity, as well as challenges in controlling the adverse effects, should be considered in decision making (4–11). Short-term adverse effects typically occur during the treatment and usually resolve within months of the completion of therapy; these adverse effects include emesis, nausea, stomatitis, myelosuppression, myalgia, and alopecia. Long-term adverse effects might have a delayed onset and sustained impact, often lasting for many years (7). In contrast, although the neoadjuvant endocrine therapy (NET) is not yet considered as a standard of care in premenopausal women and should be studied in the context of a clinical trial, the therapy could be an alternative treatment option because the adverse effects and their negative impact on quality of life (QoL) associated with endocrine therapy are relatively mild compared to those with chemotherapy for breast cancer (12–14). However, although the overall impact of ET-induced adverse effects is relatively milder than those of cytotoxic chemotherapy, these adverse effects, such as hot flashes and mood disorders, also affect the QoL of the patients and might lead to discontinuation of the therapy (15–19).

These adverse effects may cause physiological and emotional changes that could affect the patients’ QoL. Some studies reported that young patients with breast cancer might have a poorer QoL than older patients because of the distinct impact on their physical and psychosocial well-being (20–22). Improving the ability to predict an individual woman’s risk of both long- and short-term adverse effects with various treatments will help her make a better-informed decision regarding therapy. More importantly, the impact of therapy-related adverse effects on young women with breast cancer has not been adequately evaluated.

In our phase III study among premenopausal patients with hormone-responsive, human epidermal growth factor receptor-2 (HER2) negative, lymph node-positive breast cancer [NEST] (NCT01622361) (23), the efficacy, safety, and patient-reported outcomes (PROs) of NET were compared with that of NCT. This study aimed to evaluate short-term treatment-related outcomes using breast cancer-specific PROs from the NEST trial. We hypothesized that different treatment types, namely NCT or NET, would have different impacts on QoL.

## Materials and Methods

### Study Design and Participants

The NEST study was a prospective, multicenter, randomized, parallel-group, comparative phase III clinical trial. Seven centers attached to the Korean Breast Cancer Society Group participated in this study (KBCSG-012). This study protocol was approved by the Korea Food and Drug Administration as well as the institutional review board of every trial center and was conducted in accordance with the Declaration of Helsinki, good clinical practice, and the applicable local regulatory requirements on bioethics.

**Figure 1:**
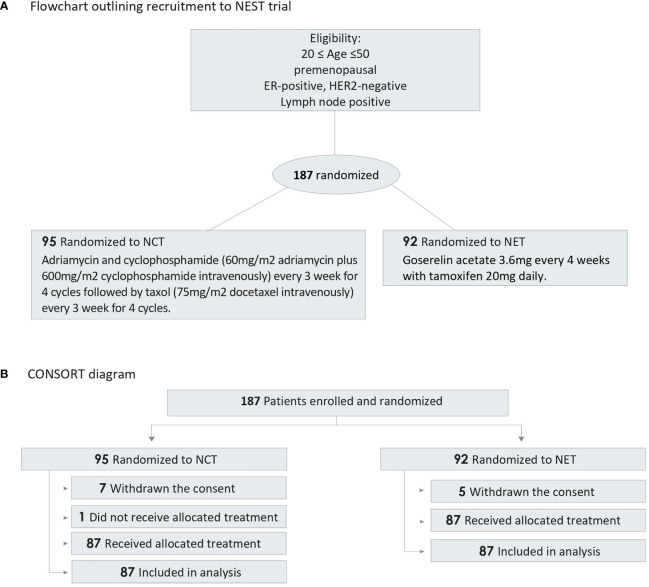
Flowchart and CONSORT Diagram. ER, estrogen receptor; HER2, human epidermal growth factor receptor 2; NCT, neoadjuvant chemotherapy; NET, neoadjuvant endocrine therapy. **(A)** Flowchart outlining recruitment to NEST trial, **(B)** CONSORT diagram of participant randomization.

Patients were randomly assigned (1:1) to receive either adriamycin and cyclophosphamide (60 mg/m^2^ adriamycin plus 600 mg/m^2^ cyclophosphamide intravenously) every 3 weeks for four cycles followed by taxol (75 mg/m^2^ docetaxel intravenously) every 3 weeks for four cycles, or gonadotropin-releasing hormone agonist (3.6 mg) every 4 weeks with tamoxifen 20 mg daily. The treatment was continued for 24 weeks before surgery (**Figure 1** and **Supplement 1**).

### PRO Assessments

PROs were assessed using the European Organisation for Research and Treatment of Cancer Quality of Life Questionnaire Core Breast Cancer Module 23 (EORTC QLQ-BR23, version 3.0) on day 1 (baseline) and at the end of treatment. The EORTC QLQ-BR23 is a breast cancer-specific module comprising four functional scales and four symptom scales. Responses to all items were converted to a 0 to 100 scale using a standard scoring algorithm (24). For functional scales, the higher scores represent a better level of functioning and QoL. For symptom scales, a higher score represents a greater severity in the symptoms. Hence, a negative change from baseline in the symptom scales reflects an improvement and a positive change reflects a deterioration. Conversely, a negative change from baseline in the functional scales reflects a deterioration, and a positive change reflects an improvement. Hair loss and alopecia were evaluated according to CTCAE Ver 5.0 (25).

### Statistical Analyses

Descriptive statistics and graphical methods were used to describe the degree of change in the EORTC QLQ-BR23 scores at baseline and follow-up. Higher scores indicated better functioning or higher symptom severity. The main analysis was based on the changes from baseline for EORTC QLQ-BR23 scales. To compare between the two groups, Mann-Whitney test was used. The means of difference between two groups were presented as a forest plot. A two-sided *p*<0.05 was considered statistically significant. All analyses were conducted using SAS version 9.4 (SAS Institute; Cary, NC) and R version 3.6.1.

## Results

Between July 5, 2012, and May 30, 2017, a total of 187 patients from seven participating centers were included and randomly allocated to one of the two treatment arms. Seven patients in the NCT group and five patients in the NET group withdrew their consent. One patient who was randomly allocated to the NCT group did not receive the treatment. Therefore, a total of 174 patients completed the scheduled treatment and were finally analyzed (87 patients received NCT and 87 patients received NET). Patient characteristics and consort diagram are shown in **Table 1** and **Figure 1**, respectively.

**Table 1:** Patient demographics and baseline characteristics.

	NCT group (n=87)	NET group (n=87)	*p* value
**Age**			0.255
Mean (SD)	42.5 ± 5.6	41.5 ± 5.8	
20-29	2 (2.3%)	2 (2.3%)	
30-39	20 (23.0%)	31 (35.6%)	
40-49	59 (69.0%)	50 (59.8%)	
50-55	6 (5.7%)	4 (2.3%)	
**BMI (kg/m2)**			0.921
<18.5	5 (5.7%)	4 (4.6%)	
18.5-24.9	54 (62.1%)	56 (64.4%)	
25-29.9	28 (27.6%)	27(23.0%)	
≥30	4 (4.6%)	7 (8.0%)	
**Clinical T stage**			0.746
T1	13 (14.9%)	9 (10.3%)	
T2	58 (66.7%)	62 (71.3%)	
T3	16 (18.4%)	16 (18.4%)	
**Clinical N stage**			0.808
N1	78 (89.7%)	76 (87.4%)	
N2	5 (5.7%)	5 (5.7%)	
N3	4 (4.6%)	6 (6.9%)	
**Grade**			0.616
G1/2	52 (59.8%)	61 (70.1%)	
G3	3 (3.4%)	4 (4.6%)	
N/A	32 (36.8%)	22 (25.3%)	
**Ki 67 expression (%)**			0.891
≤20%	49 (56.3%)	48 (55.2%)	
>20%	36 (41.4%)	37 (42.6%)	
Unknown	2 (2.3%)	2 (2.3%)	
**Planned operation**			0.141
Mastectomy	45 (51.7%)	53 (60.6%)	
Breast Conserving Surgery	42 (48.3%)	34 (39.1%)	

Data are n (%), unless otherwise stated.

NCT, neoadjuvant chemotherapy; NET, neoadjuvant endocrine therapy; SD, standard deviation.

The PROs analysis showed the results of the functional scales and symptom scales. The sample sizes for scores related to “sexual enjoyment” and the “upset by hair loss” scales were considerably smaller than those for the other symptom scales because these questions were only answered if the patients responded that they were sexually active (sexual enjoyment) and/or if the patient experienced hair loss (upset by hair loss), respectively.

### Patient-Reported Functional Scales (QLQ-BR23)

The mean baseline scores of the functional scales (NCT *vs.* NET) of body image (80.69 *vs.* 83.21), sexual function (21.23 *vs.* 20.24), sexual enjoyment (37.93 *vs.* 40.23), and future perspective (45.88 *vs.* 36.40) were generally similar, and the differences were not statistically significant between the treatment groups (**Table 2**).

**Table 2:** Baseline and follow-up EORTC QLQ-BR23 scores.

	NCT group (n=87)			NET group (n=87)			*p* value
	n	mean	95% CI	n	mean	95% CI	
**Baseline**							
**Functional scales** ^a^							
Body image	85	80.69	(76.27, 85.1)	87	83.21	(79.38, 87.03)	0.457
Sexual functioning	84	21.23	(16.8, 25.66)	84	20.24	(15.81, 24.66)	0.775
Sexual enjoyment	29	37.93	(31.39, 44.47)	29	40.23	(31.67, 48.79)	0.905
Future perspective	85	45.88	(39.14, 52.63)	87	36.40	(29.75, 43.04)	0.055
**Symptom scales/items** ^b^							
Systemic therapy side effects	85	19.66	(16.73, 22.59)	87	19.38	(17.03, 21.72)	0.639
Breast symptoms	85	21.86	(18.29, 25.44)	87	22.51	(18.92, 26.1)	0.870
Arm symptoms	85	19.61	(16.09, 23.13)	87	20.82	(16.83, 24.8)	0.890
Upset by hair loss	38	42.98	(32.82, 53.14)	34	32.35	(20.73, 43.98)	0.104
**Follow up**							
**Functional scales** ^a^							
Body image	80	68.54	(62.02, 75.06)	80	70.21	(64.6, 75.81)	0.942
Sexual functioning	80	12.92	(9.28, 16.55)	78	11.54	(7.74, 15.34)	0.468
Sexual enjoyment	19	38.60	(30.54, 46.65)	16	37.50	(26.5, 48.5)	0.903
Future perspective	80	37.92	(30.76, 45.07)	80	42.92	(36.32, 49.51)	0.309
**Symptom scales/items** ^b^							
Systemic therapy side effects	80	41.33	(36.09, 46.57)	80	36.80	(31.79, 41.8)	0.201
Breast symptoms	80	16.98	(13.62, 20.34)	80	14.48	(10.93, 18.03)	0.159
Arm symptoms	80	34.31	(29.26, 39.35)	80	28.75	(23.72, 33.78)	0.101
Upset by hair loss	51	45.10	(34.69, 55.51)	46	44.93	(35.32, 54.54)	0.891
**Difference**							
Functional scales*							
Body image	80	-13.44	(-21.21, -5.67)	80	-12.36	(-19.62, -5.1)	0.851
Sexual functioning	79	-9.07	(-14.98, -3.16)	75	-9.78	(-14.86, -4.69)	0.678
Sexual enjoyment	6	0.00	(-22.12, 22.12)	9	-11.11	(-33.3, 11.08)	0.462
Future perspective	80	-8.75	(-19.02, 1.52)	80	8.33	(-1.72, 18.38)	0.021
**Symptom scales/items**							
Systemic therapy side effects	80	21.51	(15.81, 27.2)	80	17.75	(12.3, 23.19)	0.294
Breast symptoms	80	-3.75	(-8.31, 0.81)	80	-7.71	(-12.49, -2.93)	0.438
Arm symptoms	80	15.00	(9.27, 20.73)	80	8.61	(1.75, 15.47)	0.352
Upset by hair loss	23	1.45	(-20.91,23.81)	17	15.69	(-8.64,40.01)	0.557

*Mann-Whitney test.

a Larger values indicate improvement.

b Larger values indicate deterioration.

CI, confidence interval; EORTC, European Organisation for Research and Treatment of Cancer; NCT, neoadjuvant chemotherapy; NET, neoadjuvant endocrine therapy; QLQ-BR23, Quality of Life Questionnaire Breast Cancer Module.

**Figure 2:**
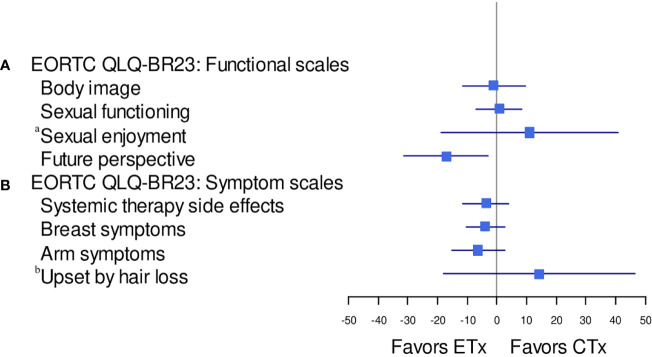
Forest plot model of estimated difference (NET: ETx *vs.* NCT: CTx) in overall change from baseline (repeated-measures mixed-effect model) in PRO-evaluable population. CTx, chemotherapy; EORTC, European Organisation for Research and Treatment of Cancer; ETx, endocrine therapy; NCT, neoadjuvant chemotherapy; NET, neoadjuvant endocrine therapy; PRO, patient-reported outcome; QLQ-BR23, Quality of Life Questionnaire Breast Cancer Module; QoL, quality of life. **(A)** EORTC QLQ-BR23: functional scales, **(B)** EORTC QLQBR23 symptom scales. ^a^The sample sizes for the "sexual enjoyment" functional scale were smaller than other functional scales because patients were asked to respond question that they were sexually active. ^b^The sample sizes for the "upset by hair loss" symptom scale were smaller than other symptom scales because patients were asked to respond to this question only if they responded in a previous question that they were experiencing hair loss.

However, a statistically significant and greater overall change from baseline favoring the NET group than in the NCT group was observed in the score for future perspective (**Figure 2**). The mean change from baseline for future perspective decreased by 8.75 in the NCT group and increased by 8.33 in the NET group, and the difference between the two groups was statistically significant (p=0.021) (**Figure 2**).

**Figure 3:**
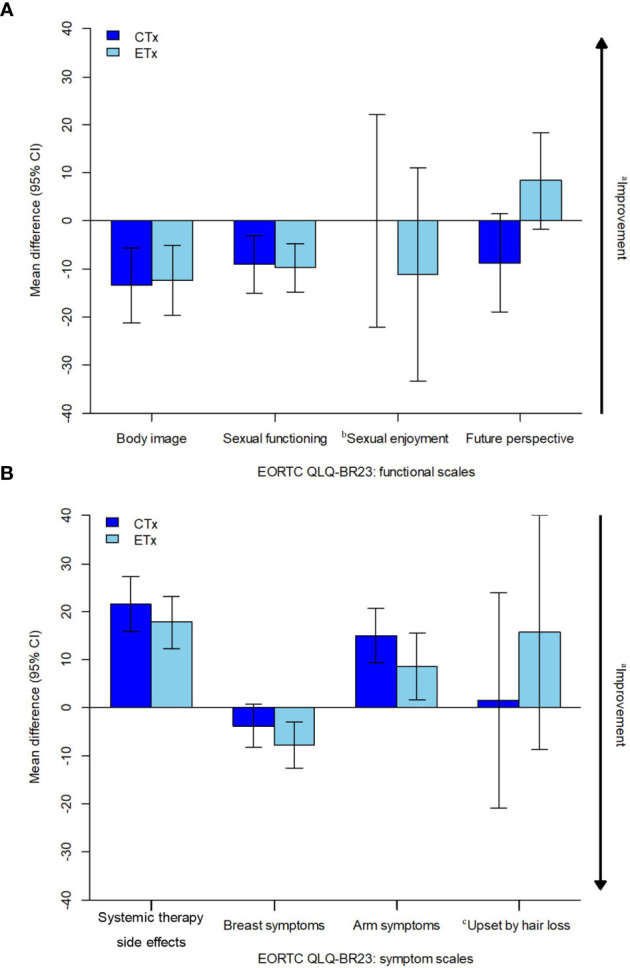
Estimated overall change from baseline in PRO-evaluable population. CI, confidence interval; CTx, chemotherapy; EORTC, European Organisation for Research and Treatment of Cancer, ETx, endocrine therapy; NCT, neoadjuvant chemotherapy; NET, neoadjuvant endocrine therapy; PRO, patient-reported outcome; QLQ-BR23, Quality of Life Questionnaire breast cancer module; QoL, quality of life. **(A)** EORTC QLQ-BR23: functional scales, **(B)** EORTC QLQ-BR23: symptom scales. ^a^Arrow denotes direction of improved outcome. ^b^The sample sizes for the "sexual enjoyment" functional scale was smaller than other functional scales because patients were asked to respond to this question only if they responded in a previous question that they were sexually active. ^c^The sample sizes for the ‘upset by hair loss’ symptom scale was smaller than other symptom scales because patients were asked to respond to this question only if they responded in a previous question that they were experiencing hair loss.

No statistically significant differences between the groups were observed in the functional scales for body image, sexual function, and sexual enjoyment (**Figure 3A**).

### Patient-Reported Symptom Scales (QLQ-BR23)

The mean baseline scores of the symptom scales (NCT *vs.* NET) of systemic therapy-related adverse effects (19.66 *vs.* 19.38), breast symptoms (21.86 *vs.* 22.51), and arm symptoms (19.61 *vs.* 20.82) were similar between both the treatment arms except for “upset by hair loss,” which was considerably lower in the NET arm (42.98 *vs.* 32.35). The question was to be answered only if the patient experienced hair loss; hence, the sample size for the “upset by hair loss” symptom was relatively small (NCT, 23; NET, 17) compared to that for other symptoms (**Table 2**).

No statistically significant differences between the groups were observed in the symptom scales for systemic therapy-related adverse effects, breast symptoms, and arm symptoms (**Figures 2**, **3B**). Grade 2 alopecia, which is defined as hair loss of ≥50% normal for that individual that is readily apparent to others, according to CTCAE Ver 5.0 (25), was not reported in the endocrine group; however, a greater overall change from baseline in the symptom scale for “upset by hair loss” was observed in the NET arm than in the NCT arm; nevertheless, this difference was not statistically significant (**Figures 2**, **3B**). The mean change from baseline score for “upset by hair loss” increased by 1.45 in the NCT group and 15.69 in the NET group; however, the difference between the two groups was not statistically significant. (p=0.557) (**Figures 2**, **3B**).

**Figure 4:**
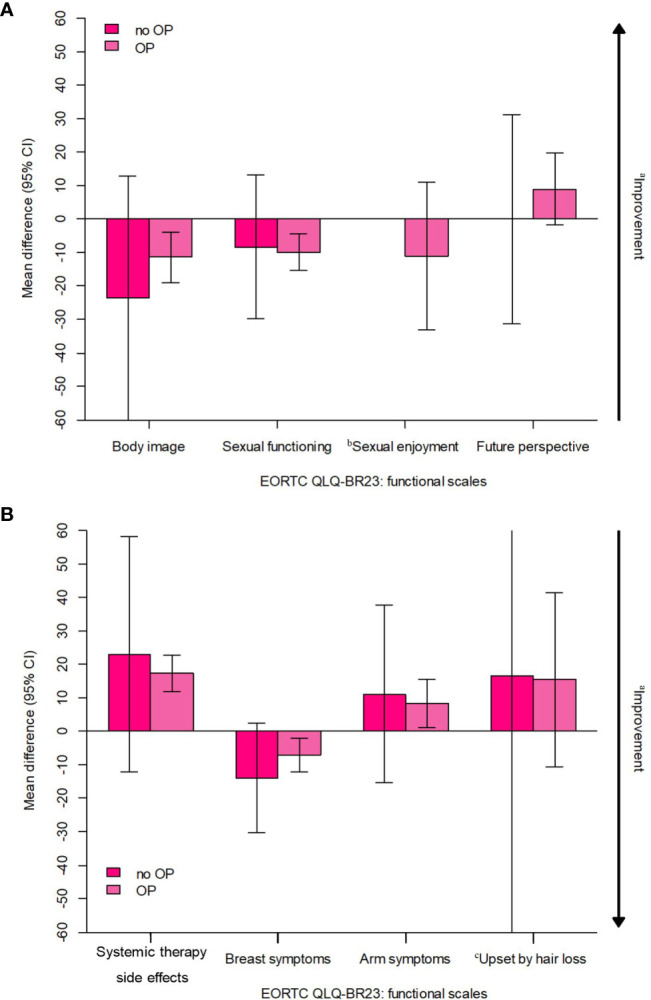
Estimated overall change from baseline in 6 patients who refuse to undergo surgery after treatment (all received NET). CI, confidence interval; EORTC, European Organisation for Research and Treatment of Cancer; NET, neoadjuvant endocrine therapy; PRO, patient-reported outcome; QLQ-BR23, Quality of Life Questionnaiure Breast Cancer Module; QoL, quality of life. **(A)** EORTC QLQ-BR23; functional scales, **(B)** EORTC QLQ-BR23: symptom scales. ^a^Arrow denotes direction of improved outcome. ^b^The sample sizes for the "sexual enjoyment" functional scale was smaller than other fuctional scales because patients were asked to respond to this question only if they responded in a previous question that they were sexually active. ^c^The sample sizes for the "upset by hair loss" symptom scale was smaller than other symptom scales because patients were asked to respond to this question only if they responded in a previous question that they were experiencing hair loss.

Among the 87 NET patients, six refused to undergo surgery after treatment. These six patients showed worse scores compared to the other patients in body image functional scales, systemic therapy-related adverse effects, arm symptoms, and “upset by hair loss” symptoms scales; however, none of them were statistically significant (**Figure 4**).

## Discussion

We have presented the first detailed cancer-related and breast cancer-specific PROs of a randomized clinical trial comparing NCT *vs.* NET in premenopausal patients with estrogen receptor-positive and HER2-negative, lymph node-positive breast cancer. In both treatment groups, no statistically significant differences were observed between the baseline and post-treatment scores in the overall PROs, including functional scales and symptom scales, except for “future perspective,” which was better in the NET group than in the NCT group. However, in the study conducted by Ferreira et al, although it was an adjuvant setting, future perspective recovery was smaller among the groups treated with endocrine therapy (26). This means endocrine therapy seems to attenuate the recovery in domains that typically improve over time such as emotional function and future perspectives. In contrast, the impact of chemotherapy seemed to be transient. These findings suggest long-term follow-up for our study group which may lead to different findings compare to current results.

In general, 15–33% of patients with breast cancer experience concerns related to body image, according to a cross‐sectional study by Falk Dahl et al. (27). In our study, the scores for “body image” dropped by more than 10 points from the baseline to post-treatment regardless of the treatment type. This difference is very important, as mean differences of 10 points or more have been considered clinically significant (28). Considering that body image is significantly correlated with adverse psychosocial consequences, such as depression (29) and poor QoL (30), physicians should monitor the distress related to altered appearance and help breast cancer patients cope with the related problems not only during the treatment period but also after completion of treatment.

In this study, the overall post-treatment scores in the symptom scales were similar in both treatment groups, including systemic therapy-related adverse effects, breast symptoms, arm symptoms, and “upset by hair loss” symptoms. Notably, only the scores of “breast symptoms” in the symptom scales were increased in both treatment arms, which indicated an improvement. This might be due to the relief derived from the treatment. Although there was no grade 2 alopecia in the endocrine group, post-treatment scores for “upset by hair loss” were similar in both groups. This result is unusual as it would usually be expected that patients in the chemotherapy arm would report higher rates of ‘‘upset by hair loss’’. In this section, we analyzed both baseline and follow-up answers and also did an analysis with a policy that for assumed a “not at all” category for women who did not answer, and it showed a similar result (**Supplementary Table 1**). This might be due to the patients’ awareness and preparedness for chemotherapy-induced alopecia, which is a well-known adverse effect. Whereas in NET, hair loss is generally considered as an uncommon adverse effect, so if NET patients experience hair loss, it would be more disappointing. Among the adverse effects induced by therapies, hair loss or hair thinning has one of the highest negative effect on the QoL in patients (31, 32). Although hair thinning or hair loss induced by therapies is a temporary effect, it might cause considerable psychological and emotional distress in patients with breast cancer. While physicians often consider skin reactions such as hair loss as relatively minor compared to the other adverse effects, patients report a higher concern about the dermatological toxicity from anti-cancer therapy (31). These negative effects might lead to the discontinuation of treatment, and indeed some patients might refuse chemotherapy only because of the alopecia (33). Endocrine therapy might also cause hair loss due to the anti-androgenic effects of the therapy, although the reported incidence of high-grade alopecia with endocrine therapy is relatively low compared with chemotherapy (34, 35). Endocrine therapy (Tamoxifen or gonadotropin-releasing hormone agonist) in hormone receptor-positive breast cancer patients reduces the estrogen levels and might cause hair loss or thinning (36). Freites-Martinez et al. (37) reported that patients receiving endocrine therapies might develop pattern alopecia similar to an androgen type, consistent with the mechanism of action of the causal agents. In a meta-analysis of 35 trials, Saggar et al. (34) reported that the overall incidence of ETs-induced alopecia was 4.4% and ranged from 0% to 25.4%, with the highest incidence in tamoxifen-treated patients. Gallicchio et al. (38) reported that approximately 25% of the patients receiving endocrine therapy experienced hair loss or thinning, and similar incidences of flushes and arthralgia related to endocrine therapy, which are known to affect the QoL (39, 40).

This relatively unexpected and disappointing outcome could have been mitigated by counseling and detailed education by the physicians about the adverse effects, especially hair loss or thinning, before the initiation of treatment (31, 41). However, it should be emphasized that emotional and psychological support to manage the impact of the adverse effect is also crucial, especially in patients receiving NET, in which hair loss or hair thinning is generally considered as unexpected or underrated compared to patients undergoing NCT. Studies have shown the effect of intervention or education in managing the adverse effects in patients receiving chemotherapy (42–46). Bourmaud et al. (47) showed promising efficacy for the educational program to improve treatment adherence and side effect management. Blanckenburg et al. (48) showed that the optimization of expectations might be a potential pathway in health care to improve patients’ QoL. Recently, Jacobs et al, designed a randomized controlled trial that employs a patient-centered, evidence-based, virtual videoconference intervention to reduce the impact of adverse effects and to improve adherence to adjuvant endocrine therapy as well (49).

This educational and emotional support might have a positive influence on patients undergoing NET by emphasizing that NET-induced hair loss or hair thinning could be more distressing than expected and encourage the patients to be prepared for the impact.

Limitations of this study include that the QoL assessment was conducted only using the EORTC QLQ-BR23 tool. QoL of the studied patient population could have been further assessed by the World Health Organization Quality of Life-BREF questionnaire, and subsequent comparison with the EORTC QLQ-BR23 could have been valuable and would have strengthened the QoL information of the current study. Other limitations are the small sample size in the “upset by hair loss” section (38 and 34 patients) which results in a major comparison limitation. These factors could have caused the bias on “upset by hair loss” in symptom scale. In addition, in this study, we only measure one time of follow-up at six months, and the perception of “upset by hair loss’’ and all other domains of symptom scales may change over time. And adjuvant endocrine therapy persists for years, thus, it would be much better to analyze the time to deterioration (TTD) in symptom and functional scales based on the median time for treatment side effects to appear. Further research on QoL in a larger patient population might help clinicians to further understand the true impact on the QoL of the patients.

In conclusion, overall PROs were similar in both treatment groups, except for “future perspective” in the functional scales of EORTC QLQ-BR23 which was significantly better in the NET group than in the NCT group. The result provides a clinical rationale to emphasize pre-treatment education or emotional support, including that for expected effects on hair, to patients receiving NET as well as those undergoing NCT.

## Data Availability Statement

The raw data supporting the conclusions of this article will be made available by the authors, without undue reservation.

## Ethics Statement

The studies involving human participants were reviewed and approved by Asan Medical Center Institutional Review Board. The patients/participants provided their written informed consent to participate in this study. Written informed consent was obtained from the individual(s) for the publication of any potentially identifiable images or data included in this article.

## Author Contributions

HK designed the study. SG and HK drafted the manuscript and HK wrote the original protocol for the study. All authors participated in the design of the study. HK filed for ethical approval from the Korea Food and Drug Administration and registered the trial on clinicaltrials.gov. GG was responsible for the pathology reports. SK-O performed the statistical analysis. SA conceived of the study and participated in its design. SA, WN, EL, YJ, LK, WH, and SN were involved in the study design and inclusion of patients in this trial. All authors contributed to the article and approved the submitted version.

## Funding

This study was sponsored by AstraZeneca Korea Ltd. The funder was not involved in the study design, collection, analysis, interpretation of data, the writing of this article or the decision to submit it for publication.

## Conflict of Interest

The authors declare that the research was conducted in the absence of any commercial or financial relationships that could be construed as a potential conflict of interest.
