# Supplementary material for: Patient-Reported Outcomes From Phase III Neoadjuvant Systemic Trial Comparing Neoadjuvant Chemotherapy With Neoadjuvant Endocrine Therapy in Pre-Menopausal Patients With Estrogen Receptor-Positive and HER2-Negative, Lymph Node-Positive Breast Cancer
Source: Front Oncol. 2021 Jul 2;11:608207. doi: 10.3389/fonc.2021.608207 (PMC8284076; doi:10.3389/fonc.2021.608207)
Supplement: Supplementary file 1 [file Table_1.docx]

**Supplementary Table 1. Baseline and follow-up EORTC QLQ-BR23 scores**

**(including one sided answer for “upset by hair loss, baseline only or follow-up only)**

|  | NCT group (n=87) | | | NET group (n=87) | | |  | |
| --- | --- | --- | --- | --- | --- | --- | --- | --- |
|  | n | mean | 95% CI | n | mean | 95% CI | *p* value | |
| **Baseline** |  |  |  |  |  |  |  | |
| **Functional scales**^a^ |  |  |  |  |  |  |  | |
| Body image | 85 | 80.69 | (76.27, 85.1) | 87 | 83.21 | (79.38, 87.03) | 0.457 | |
| Sexual functioning | 84 | 21.23 | (16.8, 25.66) | 84 | 20.24 | (15.81, 24.66) | 0.775 | |
| Sexual enjoyment | 29 | 37.93 | (31.39, 44.47) | 29 | 40.23 | (31.67, 48.79) | 0.905 | |
| Future perspective | 85 | 45.88 | (39.14, 52.63) | 87 | 36.40 | (29.75, 43.04) | 0.055 | |
| **Symptom scales/items**^b^ |  |  |  |  |  |  |  | |
| Systemic therapy side effects | 85 | 19.66 | (16.73, 22.59) | 87 | 19.38 | (17.03, 21.72) | 0.639 | |
| Breast symptoms | 85 | 21.86 | (18.29, 25.44) | 87 | 22.51 | (18.92, 26.1) | 0.870 | |
| Arm symptoms | 85 | 19.61 | (16.09, 23.13) | 87 | 20.82 | (16.83, 24.8) | 0.890 | |
| Upset by hair loss | 38 | 42.98 | (32.82, 53.14) | 34 | 32.35 | (20.73, 43.98) | 0.104 | |
| **Follow up** |  |  |  |  |  |  |  | |
| **Functional scales**^a^ |  |  |  |  |  |  |  | |
| Body image | 80 | 68.54 | (62.02, 75.06) | 80 | 70.21 | (64.6, 75.81) | 0.942 | |
| Sexual functioning | 80 | 12.92 | (9.28, 16.55) | 78 | 11.54 | (7.74, 15.34) | 0.468 | |
| Sexual enjoyment | 19 | 38.60 | (30.54, 46.65) | 16 | 37.50 | (26.5, 48.5) | 0.903 | |
| Future perspective | 80 | 37.92 | (30.76, 45.07) | 80 | 42.92 | (36.32, 49.51) | 0.309 | |
| **Symptom scales/items**^b^ |  |  |  |  |  |  |  | |
| Systemic therapy side effects | 80 | 41.33 | (36.09, 46.57) | 80 | 36.80 | (31.79, 41.8) | 0.201 | |
| Breast symptoms | 80 | 16.98 | (13.62, 20.34) | 80 | 14.48 | (10.93, 18.03) | 0.159 | |
| Arm symptoms | 80 | 34.31 | (29.26, 39.35) | 80 | 28.75 | (23.72, 33.78) | 0.101 | |
| Upset by hair loss | 51 | 45.10 | (34.69, 55.51) | 46 | 44.93 | (35.32, 54.54) | 0.891 | |
| **Difference*** |  |  |  |  |  |  |  | |
| **Functional scales** |  |  |  |  |  |  |  | |
| Body image | 80 | -13.44 | (-21.21, -5.67) | 80 | -12.36 | (-19.62, -5.1) | 0.851 | |
| Sexual functioning | 79 | -9.07 | (-14.98, -3.16) | 75 | -9.78 | (-14.86, -4.69) | 0.678 | |
| Sexual enjoyment | 32 | -17.71 | (-30.65, -4.76) | 30 | -21.11 | (-33.55, -8.67) | 0.825 | |
| Future perspective | 80 | -8.75 | (-19.02 ,1.52) | 80 | 8.33 | (-1.72, 18.38) | 0.021 | |
| **Symptom scales/items** |  |  |  |  |  |  |  | |
| Systemic therapy side effects | 80 | 21.51 | (15.81 ,27.2) | 80 | 17.75 | (12.3, 23.19) | 0.294 | |
| Breast symptoms | 80 | -3.75 | (-8.31 ,0.81) | 80 | -7.71 | (-12.49, -2.93) | 0.438 | |
| Arm symptoms | 80 | 15.00 | (9.27 ,20.73) | 80 | 8.61 | (1.75, 15.47) | 0.352 | |
| Upset by hair loss | 63 | 13.23 | (-0.47, 26.93) | 59 | 19.21 | (7.33, 31.09) | 0.596 | |
| *Mann-Whitney test  *baseline hair loss (-) follow-up hair loss (+) = not at all  *baseline hair loss (+) follow-up hair loss (-) = not at all  ^a^Larger values better.  ^b^Larger values worse. | | | | | | | |  |

CI, confidence interval; EORTC, European Organisation for Research and Treatment of Cancer; NCT, neoadjuvant chemotherapy; NET, neoadjuvant endocrine therapy; QLQ-BR23, Quality of Life Questionnaire Breast Cancer Module
